# When There Is Not Enough Evidence and When Evidence Is Not Enough: An Australian Indigenous Smoking Policy Study

**DOI:** 10.3389/fpubh.2016.00228

**Published:** 2016-10-20

**Authors:** Daniel Vujcich, Mike Rayner, Steven Allender, Ray Fitzpatrick

**Affiliations:** ^1^Western Australian Department of Health, Public Health Division, Perth, WA, Australia; ^2^Nuffield Department of Population Health, University of Oxford, Oxford, UK; ^3^School of Health and Social Development, Deakin University, Geelong, VIC, Australia

**Keywords:** aboriginal health, policy making, evidence-based policy, tobacco, indigenous health, policy analysis, smoking cessation

## Abstract

**Background:**

The *Indigenous Tobacco Control Initiative* and *Tackling Indigenous Smoking Measure* were both announced by the Australian Government at a time when its rhetoric around the importance of evidence-based policy making was strong. This article will (1) examine how the Rudd Government used evidence in Indigenous tobacco control policy making and (2) explore the facilitators of and barriers to the use of evidence.

**Methods:**

Data were collected through (1) a review of primary documents largely obtained under the *Freedom of Information Act 1982* (Commonwealth of Australia) and (2) interviews with senior politicians, senior bureaucrats, government advisors, Indigenous health advocates, and academics. Through the *Freedom of Information Act* process, 24 previously undisclosed government documents relevant to the making of Indigenous tobacco control policies were identified. Interviewees (*n* = 31, response rate 62%) were identified through both purposive and snowball recruitment strategies. The Framework Analysis method was used to analyze documentary and interview data.

**Results:**

Government policy design was heavily influenced by the recommendations presented in government authored/commissioned literature reviews. Resulting policies were led by equivocal evidence for improved tobacco control outcomes among Indigenous Australians. Many of the cited studies had methodological limitations. In the absence of high-quality evidence, some policy makers supported policy recommendations that were perceived to be popular among the Indigenous community. Other policy makers recognized that there were barriers to accumulating rigorous, generalizable evidence; in the absence of such evidence, the policy makers considered that the “need for action” could be combined with the “need for research” by introducing innovative strategies and evaluating them.

**Discussion:**

Despite the absence of high-quality evidence, the formulation and adoption of Indigenous tobacco policy was neither irrational nor reckless. The decision to adopt an innovate and evaluate strategy was justifiable given (a) the potential for the gap between Indigenous and non-Indigenous health outcomes to worsen in the absence of an imminent policy response; (b) the existence of circumstances, which made it difficult to obtain high-quality evidence to guide policy; and (c) the need for policy solutions to reflect community preferences, given sociohistorical sensitivities.

## Introduction

“Evidence-based policy” has become a synonym for “good policy,” prompting the observation that “[i]t is difficult to imagine anyone arguing that policy should be based on anything but the best available evidence” (1). In a critical analysis of literature relating to the use of evidence in health policy making, Oliver et al. noted that the majority of studies made normative assumptions that policies *should* be based on evidence, and that any barriers between evidence and policy ought to be overcome (2). However, few of the studies used empirical data to demonstrate *why* evidence-based policy making is superior to other forms of decision-making. Similarly, only a small number of studies provided in-depth empirical descriptions of the importance of evidence relative to other considerations.

Numerous models and theories conceptualize evidence as just one part of a broader scheme of competing influences on policy. For instance, Walt and Gilson considered that the *content* of health policies is affected by an interplay between *context* (e.g., culture, economic factors, demography, history, ideology), *actors* (i.e., individuals, groups, or organizations and their position in the power hierarchy), and the *process* through which policy issues are communicated, negotiated, and decided (which can include the consideration of evidence) (3, 4).

A contemporary adaptation of this model is Lin’s description of health policy as the product of three competing rationalities (5). “Cultural rationality” is defined as “values, ethics, what (perceived) societal opinions feel is right in relation to health policy” and thus aligns with what Walt and Gilson term “context” (5). “Political rationality” relates to the process through which power is exercised and decisions are made and includes such factors as “the willingness of policymakers to have transparent processes and be accountable, the ability of interest groups to participate … and the role of commentators (be it media, experts, or lobbyists)” (5). Finally, “technical rationality” describes the knowledge produced by researchers and can include diverse forms of evidence, such as epidemiology and economics (5). Lin argues that these rationalities (and the policies that they create) are shaped by “historical political legacies” and reflect “ongoing processes of social learning” (5).

In view of such rich theoretical explanations, Oliver et al. call for a new research agenda, which focusses on the “influences on and processes of policy” through in-depth, empirical descriptions of how evidence “fits with the other drivers and triggers that affect policy” (2). This article uses the development of tobacco control policies for Indigenous Australians as a case study for examining the real-world tensions inherent in the health policy-making process.

When the Labor Party assumed government in 2007 under the leadership of Prime Minister Kevin Rudd, Indigenous Australians were expected to have lives that were 17 years shorter than their non-Indigenous counterparts (6). The Labor Party argued that such enduring disparities were a product of ineffective, ideologically driven policies and that “from here on our guiding principle will be the evidence of what works and what does not work in reducing disadvantage” (7).

The *Indigenous Tobacco Control Initiative* and *Tackling Indigenous Smoking Measure* committed over AUD 120 million in government funds and marked the beginning of a period “that has seen more action on Indigenous smoking than any other time in our history” (8). Yet, perhaps surprisingly, in light of the foregoing context, it was said of some aspects of the policies that “the government hasn’t produced evidence to back up its campaign” (9).

This article will (1) examine how the Rudd Government used evidence in Indigenous tobacco control policy making and (2) explore the facilitators of and barriers to the use of evidence relative to other factors.

In so doing, this article reveals difficulties in the application of evidence-based policy making. These difficulties include debates as to what constitutes “evidence” and a tension between the need for good evidence and the need for urgent policy action. The article also demonstrates how, in some circumstances, the need for evidence of effectiveness can be exceeded by the need to empower the target population in the decision-making process.

## Materials and Methods

Data were collected through a combination of document reviews and interviews. The overarching purpose of data collection with respect to each method is set out in Table 1 below. Data collection and subsequent analysis were informed by awareness of the possibility that key factors might turn out to relate to “context/cultural rationality” and “political rationality/process” as much as “technical rationality.”

**Table 1:** **Summary of goals of data collection, by method**.

Method		
**Goals**
	**Document review**	**Interviews**

	Identify key policy actors to be included in the interview sample (purposive and snowballing)	
	Understand the content of relevant Indigenous tobacco control policies and aspects of how those policies were made in order to inform the interview questions	
	Obtain information to piece together an account of how the Indigenous tobacco control policies were made	
	Corroborate and fill in gaps in findings obtained through other methods of data collection	

It is considered prudent to combine these two methods when conducting policy process analyses to mitigate their respective limitations (4, 10). These limitations include the fact that (1) some important events in policy processes are often not recorded, or the documents may be withheld from public access; (2) documents can possess official and perhaps incomplete versions of events; (3) respondents may underrepresent or overrepresent their role in a policy process; and (4) the passage of time might impede a respondent’s ability to recall events accurately or with sufficient detail (10).

### Documentary Data Collection

A two-stage process was adopted to identify relevant documents. In the first instance, publicly available documents were obtained by searching the following websites/databases:

- Department of Health and Ageing (DoHA) (“Publications” and “Media Release and speech archive” sections only);
- Australian Indigenous HealthInfoNet (“Policies” and “Tobacco” sections only);
- Tobacco Control Supersite (“Australian Tobacco Timeline” section only);
- National Aboriginal and Torres Strait Islander Health Equality Council;
- National Preventative Health Taskforce;
- Australian National Council on Drugs;
- Ministerial Council on Drug Strategy;
- Intergovernmental Committee on Drugs;
- Quit;
- *Factiva* (Australian national newspapers).

Where search functions were available, the search was limited to items containing the search terms “(Aboriginal OR Indigenous) AND (tobacco OR smok*).” Where search functions were not available, items were individually screened by title for relevance. Relevant documents were limited to those produced between 2007 (election of Rudd Government) and 2008 (announcement of policies).

The second-stage of the document review was focused on obtaining relevant documents produced for the private use of members of the policy-making community. These documents were either provided by the interview respondents or accessed under the federal *Freedom of Information Act 1982* (FOI).

The initial request (FOI Request 309-112) for “written reports/letters of advice and internal reports or memoranda used by the Government to inform the design of the Indigenous Tobacco Control Initiative” resulted in an estimated charge of AUD 10,845 for the time that FOI officers and document custodians would likely spend searching for, retrieving, and assessing the documents. Further particularized requests were then submitted (FOI Requests 006-1314 and 067-1213) seeking access to the following:

- minutes of meetings held between December 2007 and 20 March, 2008 in which the *Indigenous Tobacco Control Initiative* was discussed;
- minutes of any meetings relevant to the selection of the first six multicomponent projects funded as part of the *Indigenous Tobacco Control Initiative* in 2008–09;
- minutes of meetings held between December 2007 and December 2008 in which the tobacco elements of the *National Partnership Agreement on Closing the Gap in Indigenous Health Outcomes* were discussed;
- any research commissioned before December 2008 to inform the *Tackling Smoking Measure* specifically;
- memoranda or advice prepared between December 2007 and 20 March, 2008 in which the *Indigenous Tobacco Control Initiative* is mentioned;
- memoranda, advice, or briefing papers drafted before December 2008 in which the *Tacking Indigenous Smoking Measure* is mentioned.

The revised requests yielded 24 previously undisclosed documents relevant to the making of the policies. One document was not disclosed on the basis that it was produced for Cabinet deliberations and therefore exempt under the legislation.

### Interview Data Collection

Interview participants were recruited through a combined purposive and snowballing sampling strategy, as recommended for studies of this nature (10, 11). A preliminary list of individuals involved in the policy-making process was created using the documents described above. These purposefully selected respondents were asked to name other people who influenced the policy process in relation to Indigenous tobacco control.

Fifty potential respondents were invited to participate in interviews. A source population of 50 policy actors was considered reasonable, given that the study was limited to two specific policies. Thirty-one individuals agreed to participate in the study, representing a response rate of 62%. The response rate is consistent with those obtained in other studies of the public services sector (12).

The sample comprised advisors to the Federal Minister for Health (*n* = 2), senior Federal politicians (*n* = 2), senior Federal health bureaucrats (*n* = 4), members of and assistants to the National Preventive Health Taskforce and its Tobacco Working Group (*n* = 9), members of the National Indigenous Drug and Alcohol Committee (*n* = 2), researchers/academics (*n* = 4), and Indigenous health advocates (*n* = 8). Anonymity was guaranteed to protect reputations and interests. Of those individuals who did not respond to or declined interview invitations, four were politicians, seven were government advisory group members, one was a senior bureaucrat, four were researchers, and three were Indigenous advocates.

Interviews were audio recorded and transcribed. Interviews were semi-structured, using a combination of open-ended and probing questions. Topic guides were prepared before each interview and were tailored to the knowledge and experience of each respondent. Where relevant, respondents were asked to describe the following:

- factors that might explain the policy attention given to Indigenous tobacco control under the Rudd Government;
- how the policies were developed;
- factors that might explain why certain approaches to tobacco control were prioritized over others;
- the role of evidence in the policy process;
- facilitators of and barriers to the use of evidence.

Ethics approval was granted by relevant committees at the University of Oxford (Ref: SSD/CUREC1/12-013) and Deakin University (Ref: 2012-218).

### Data Analysis

Data were analyzed using the Framework Analysis method. The process involved (1) becoming familiar with the data by listening to/transcribing the recordings and reading the transcripts and documents; (2) noting down recurring themes and subthemes (see Table 2 below); (3) using QSR International’s NVivo 10 software to code the data according to the themes; (4) transferring the coded data into charts (one for each theme), which were organized in rows by source and in columns by subtheme; and (5) looking down the chart columns to observe recurring patterns and tensions.

**Table 2:** **Themes and subthemes coded for data analysis**.

Themes	Subthemes
Political priority	Why Indigenous smoking historically not prioritized
	Factors explaining rise in prominence in 2008 Evidence Other (open coding)
Process – how policies made/adopted	Descriptions at each level National Preventative Health Taskforce Tobacco Working Group level Department of Health level Ministerial level
	Role of evidence
	Role of other factors (open coding)
Evidence	Amount available
	Quality available
	Appropriateness/applicability to policy question
	Barriers to use (open coding)
	Facilitators to use (open coding)

## Results

### The Role of Evidence in Getting Indigenous Tobacco Control on the Agenda

There had long been evidence to indicate high rates of Indigenous smoking. A 1994 survey of over 15,700 Indigenous Australians found that 49.7% of respondents over the age of 13 years smoked daily (13). Similarly, a survey conducted a decade later (2004–05) found that 50% of Indigenous Australians aged 18 years and over (*n* = 10,439) were current daily smokers, compared to 23% of the general population (14).

Despite these data, there had been an absence of significant government investment specifically geared toward reducing Indigenous smoking rates prior to 2008 (15). For instance, an expenditure analysis revealed that smoking was a focus in only 2 and 3% of Indigenous-specific alcohol and other drug intervention projects in 1999–2000 and 2006–2007, respectively (16).

The interview findings suggest that the increased political attention given to Indigenous smoking in 2008 was the product of a confluence of three factors – namely – (1) the publication of evidence showing that tobacco was the single largest contributor to the “gap” between Indigenous and non-Indigenous health outcomes; (2) the success of the *Closing the Gap* advocacy campaign, which was led by Indigenous and civil society groups and supported by the incoming Labor Government; and (3) the appointment of a Health Minister committed to tobacco control, perhaps best evidenced in the Rudd Government’s decision to make Australia the first country in the world to introduce a law to mandate plain cigarette packaging.

#### Burden of Disease Study

Six respondents cited a burden of disease study by Vos et al. published in 2007 (hereafter, the Vos study) as an explanation for why Indigenous smoking became more politically prominent in 2008 (17). The study found that, of 11 risk factors, tobacco was the largest single contributor to both the overall Indigenous disease burden (12%) and the health “gap” between Indigenous and non-Indigenous Australians (17%) measured in disability-adjusted life years.

One senior bureaucrat said of the Vos study, *“the study was so important.”* The significance of the Vos study was that it “*show[ed] people what were the contributing factors to excess mortality*” (senior bureaucrat). An Indigenous leader and health advocate, recalled that Vos’ work prompted an Indigenous research organization to focus on smoking:

*[The Cooperative Research Centre for Aboriginal and Torres Strait Islander Health] put briefings together and all that and started to tell their story to the Minister* … *And then they did this presentation in Parliament House. Within a fortnight [the Government] found $14 million*.

The presentation to which the respondent referred formed part of the Parliamentary Showcase of Aboriginal Health Research, which was held in March 2008 and opened by the Health Minister. As part of the program, Viki Briggs of the Centre for Excellence in Indigenous Tobacco Control (CEITC) delivered a presentation entitled “Reduce Smoking, Reduce the Gap” in which the findings of the Vos study were highlighted (18). Another respondent considered that *“that was probably an absolutely crucial event”* (Indigenous tobacco control academic). A Ministerial advisor considered that the event *“helped crystallise the view that tobacco was a critical factor.”*

#### The “Closing the Gap” Movement

Four respondents were of the view that the impact of the Vos study must be considered in its sociopolitical context. The study was published in 2007 at a time when the Closing the Gap campaign for Indigenous and non-Indigenous equality was gaining momentum (19). In the lead up to the 2007 Federal election, the Labor Party made a commitment to improve Indigenous social and health outcomes in response to the Closing the Gap campaign.

Evidence demonstrating the impact of smoking on the gap between Indigenous and non-Indigenous health outcomes found fertile ground in this sociopolitical landscape. According to a senior politician:

*[W]hile the strong research helped galvanise people around [Indigenous tobacco control] I don’t think it was the cause or catalyst for action* … *[T]he very successful campaign around Close the Gap* … *helped create political momentum and interest – the research is vital in this context but could’ve gone unnoticed without the broader campaign*.

Similarly, a senior bureaucrat explained that a *“catalyst”* for political action *“was that we had a figure, a statistic that said smoking causes 20% of all mortality. It came at the same time as Closing the Gap. So, okay, to close the gap* … *you’ve got to prevent premature mortality.”* A Ministerial advisor agreed that *“we were never going to achieve this [aim to close the gap in health outcomes] if we didn’t do something about Indigenous smoking.”*

#### Political Leadership

While the confluence of evidence and political agendas created a positive environment for Indigenous tobacco control to become more politically salient, four respondents emphasized the crucial role of the Health Minister in actually securing policy action. It was noted that DoHA’s interest in Indigenous tobacco control predated the policy developments in 2008 but that, despite this, the issue never gained political attention. According to a senior bureaucrat, *“[w]henever we put a case around a smoking intervention, every minister would say, ‘yeah, well who the hell’s going to run it because they [Aboriginal health staff] all smoke anyway?’”*

By contrast, the new Health Minister, Nicola Roxon, was described as someone who understood and championed the importance of Indigenous tobacco control. A Government advisor explained that the *“bottom line is Nicola Roxon* … *got it and put money into it”* (Tobacco Working Group member). Similarly, an Indigenous leader and health advocate was of the view that *“you can put it [policy action] down to a couple of people in the Minister’s office and the Minister herself. She was committed to saying, ‘if that’s what the evidence is telling us, we need to put a bigger effort into here.”*

### Role of Evidence in the Design of the *Indigenous Tobacco Control Initiative*

Both interview data and documents obtained under FOI confirmed that DoHA played a lead role in the design of the *Indigenous Tobacco Control Initiative*, which occurred over a short period of time due to political and organizational pressures. A Ministerial advisor noted “*there was a sense that after the Apology [to the Stolen Generations in February 2008] that* … *[the] goodwill created by that needed to be translated into some sort of firm commitments”* (Ministerial advisor).

The Indigenous Health Equality Summit was scheduled to take place in Parliament House in March 2008 and was expected to culminate in the signing of a partnership agreement between government and non-government organizations to achieve health equality between Indigenous and non-Indigenous Australians. The interview data suggested that the Prime Minister had expressed an intention to attend the event, and that this served as a catalyst for the development of the policy. A Ministerial advisor said:

*I do remember this being an example where we were kind of going, ‘Ergh! I think the PM might want to announce something’* … *[A]s I say, it was all a little bit chaotic in those early days. So it meant that a lot of the sort of detailed policy development was done at the Departmental level more so than at the Ministerial level*.

Media reports, to which the respondent referred, revealed that the Health Minister had been dissatisfied with unrealistic time pressures imposed by Prime Minister Rudd (20). According to the Ministerial advisor, *“the Department [of Health and Ageing] sort of came up with this bunch of proposals and said, ‘look* … *we’ll give you something to announce at this event.’”*

The *Indigenous Tobacco Control Initiative* was announced by Prime Minister Rudd at the Indigenous Health Equality Summit. Through the Initiative, the Government committed AU$14.5 million over 4 years to:

- train Indigenous health staff in smoking-cessation strategies;
- trial innovative community interventions, including culturally appropriate communication activities, in five or six pilot sites; and
- support Indigenous tobacco control research to “help build the evidence base around what works” (21).

One of the documents obtained under FOI was a literature review (dated June 2007) authored by an employee of DoHA Program Management and Evaluation Unit. The review proposed five policy options, four of which seem to have been incorporated into the Initiative developed by DoHA – namely:

- offer support and training for local health staff to deliver brief tobacco cessation interventions and programs that include nicotine replacement therapy;
- provide more intensive interventions in targeted communities/regions;
- develop media campaigns featuring local identities or artwork;
- fund the CEITC to audit evaluations of tobacco control initiatives.^1^

Table 3 below contains the studies cited in DoHA review to support each policy proposal and provides a summary of their major findings and relevant limitations.

**Table 3:** **Summary of studies cited in DoHA (see text footnote 1) review in support of policy proposals**.

Policy proposal	Supporting evidence cited in DoHA review	Type of evidence	Relevant finding(s)	Limitations
Smoking-cessation training for health staff	Ivers (22, 23)	Systematic review of published and unpublished studies on Indigenous Australian tobacco control initiatives	Review found one study by Harvey et al. (27), which used pre- and post-test interviews to evaluate a 1-day workshop that provided 34 employees in three Indigenous health-care settings with training in motivational interviewing and the stages of change model for addressing addictive behaviors Evaluation produced no evidence that any staff or clients has given up smoking at the 6-month follow-up Some evidence that training led some staff to reassess their own smoking status	Small sample Staff turnover and availability meant that pre- and post-interview participants were not identical
	Adams and Briggs (24)	Descriptive review of role of government and non-government organizations in Indigenous tobacco control	Only referred to Harvey et al. study (27) described above	
	Lindorff (25)	Survey of 67 health staff Focus groups with 275 participants from health services, legal centers, women’s centers, men’s groups, drug and alcohol rehabilitation centers, school staff, resource centers, employment programs, elders groups, and university students and staff	62% of a survey sample wanted more tobacco training Some participants in focus groups “emphasised the need for more training if staff were to deal with tobacco on a day-to-day basis… Staff training was also seen as an opportunity to prompt staff into thinking about their own smoking”	Small sample of health service staff
	Ivers et al. (26)	Pre-and post-study with matched controls Three remote Indigenous communities received government grant to implement multicomponent tobacco control interventions, and three communities were chosen as matched controls Multicomponent interventions included health worker training, as well as introduction of smoke-free public places policies, tobacco education programs, provision of nicotine patches, and point-of-sale restrictions	Found no decreases in smoking prevalence 1-year post-intervention One intervention community experienced a statistically significant decrease in the amount of tobacco consumed (based on store sales figures) after 1 year Percentage of smokers who reported thinking about quitting/taking action increased 11% after 1 year Percentage of smokers aware of the risks of lung cancer and heart disease increased 5% after 1 year	Intervention communities were self-selected Less than one quarter of residents across the intervention communities participated in surveys at both baseline and follow-up Not possible to ascertain the effects of training relative to other components of intervention
Provide intensive community interventions	Ivers et al. (26)	Pre- and post-study with matched controls (see above)	See above	See above
	Adam and Briggs (24)	Descriptive review (see above)	Describes a randomized control trial involving eight north Queensland communities with interventions including event support programs, school-based tobacco education, quit support groups, brief intervention in health services, etc. No findings from randomized controlled trial presented	N/A
Local media campaigns	Lindorff (25)	Focus groups with 275 participants from a range of groups (see above)	Focus group participants mentioned that desirable features of health promotion resources included use of Indigenous languages, Indigenous faces, pictures, simple language, video and radio media, music, interactive resources, and television advertisements featuring Indigenous people	Sampling bias – report notes that “[i]t was quite difficult to assure people that the groups were not going to be a lecture about quitting tobacco … As a result a number of potential participants chose not to take part in the groups, many of who were smokers”
	Adam and Briggs (24)	Summarizes an unpublished evaluation of a program that incorporated some culturally specific social marketing strategies (use of local Indigenous identifies) 151 Indigenous people randomly approached in street and asked exposure and recall questions	22% had heard the radio advertisement with 13% recall 10% had seen car/bus advertisement with 5% recall	Study was restricted to recall; effect on knowledge or tobacco use not discussed
	Ivers (23)	Systematic review (see above)	Review found one evaluation of a non-Indigenous anti-smoking campaign [Murphy and Mee (28)] Evaluation data obtained through 15 focus group discussions with 42 Indigenous adults, 20 Indigenous teenagers, and 23 community and health workers recruited across four locations Found that “the groups gave no indication that awareness of, or exposure to, the campaign was any different among indigenous and non-indigenous populations. Similarly, the results … gave no reason to believe that indigenous people received the campaign messages any differently to the non-indigenous population” [Murphy and Mee (28)]	Evaluation contained no description of methods by which participants were recruited and data were analyzed
Support research into Indigenous tobacco control	Ivers (22, 23)	Systematic review (see above)	Found only four evaluations of tobacco interventions for Indigenous Australians, none of which assessed smoking cessation as an outcome	N/A
	Unknown	Unpublished recommendations from a 2005 Indigenous smoking workshop convened by DoHA	Workshop recommended the development and implementation of a research program around Indigenous smoking	Unclear how recommendations developed

### Role of Evidence in the Design of the *Tackling Indigenous Smoking Measure*

The second major Indigenous tobacco control policy – the *Tackling Indigenous Smoking Measure* – formed part of the Council of Australian Governments’ *National Partnership Agreement on Closing the Gap in Indigenous Health Outcomes*, which was signed in December 2008 (29).

The interview data suggest that DoHA played a key role in the development of this policy also. According to a Ministerial advisor, “the Department developed the proposals that we kind of further refined in cooperation with them I guess.” Interviews with DoHA staff confirmed the central role of the Department in designing the policy and also highlighted the informal influence of external advisors. A senior bureaucrat recalled:

*In the early stages of [determining] ‘well, what do we do?’ we talked to the Centre for Excellence in Indigenous Tobacco Control [CEITC]* … *and we had conversations with them initially about ‘well, we’ve got money to do X and Y and Z, if you could do something what do you think needs to be done?’*

DoHA also commissioned CEITC to draft a literature review and a scoping paper to inform its response to Indigenous smoking rates. The resulting paper was provided to DoHA in September 2008.

Additionally, advice on the formulation of the policy was received from the National Preventative Health Taskforce. The National Preventative Health Taskforce was established by the Government to assist in the development of strategies to prevent diseases related to tobacco, alcohol, and obesity. While the Taskforce did not release its final recommendations until 2009, a Government report revealed that interim advice was provided in 2008 (30). According to a member of the Taskforce’s Tobacco Working Group, *“[t]hey involved us in the development of the Tackling Smoking Initiative* … *We were doing the same recommendations formally and informally.”* The Director of CEITC was a member of the Tobacco Working Group.

The formal recommendations published by the Tobacco Working Group reproduced almost verbatim the recommendations contained in the literature review and scoping paper drafted by CEITC for DoHA (31). The relevant recommendations in the CEITC paper were to

- increase the Indigenous tobacco control workforce;
- improve health worker access to appropriately designed training;
- improve access to pharmacotherapies;
- develop a well-researched and focus-tested Indigenous-specific social marketing campaign at national and local levels;
- raise the profile of Quitlines to Indigenous communities (32).

These recommendations closely aligned with the *Tackling Indigenous Smoking Measure*, which committed AUD 100.61 million to reduce Indigenous smoking rates and the burden of tobacco-related disease through a number of initiatives, the most prominent of which were the following:

- establish a national network of tobacco action coordinators;
- develop a national Indigenous tobacco action training program for health workers and community educators;
- strategies to improve delivery of smoking-cessation services, including nicotine replacement therapy;
- social marketing campaigns to reduce smoking-related harms among Aboriginal and Torres Strait Islander peoples;
- enhance Quitline to provide culturally sensitive services (29, 33).

Table 4 below summarizes the evidence cited in the CEITC paper to support each policy proposal and notes major findings and limitations of each.

**Table 4:** **Summary of studies cited in CEITC paper in support of policy proposals**.

Policy proposal	Supporting evidence cited in CEITC review	Type of evidence	Relevant finding(s)	Limitations
Specialist Indigenous tobacco control workforce	Wood et al. (34); Zandes et al. (35); Murphy and Mee (28); Mark et al. (36); Karen and Walker (37)	Qualitative studies	Generalist Indigenous health workers were reluctant to provide smoking-cessation advice because they did not want to “add to the other health and social problems and make them feel bad about themselves” and did not want to appear hypocritical on account of their own smoking status	N/A
	Market Equity (38)	Qualitative study based on interviews with 15 “key intermediaries involved in Indigenous health related to smoking”	Some respondents felt that there needed to be a team of Indigenous people working in Indigenous smoking prevention programs	Small sample Sampling strategy not clear Nature/content of interviews unclear
Tobacco action training	Lancaster and Fowler (39)	Cochrane systematic review	No strong evidence that training health professionals to provide smoking-cessation interventions has an effect on quit rates	N/A
	Harvey et al. (27)	Pre- and post-test interviews 34 Indigenous health service employees were provided with training in motivational interviewing and the stages of change model for addressing addictive behaviors	No evidence that any staff or clients has given up smoking at the 6-month follow-up Some evidence that training led some staff to reassess their own smoking status	Small sample Staff turnover and availability meant that pre- and post-interview participants were not identical
Improved access to pharmacotherapy	Stead et al. (40)	Cochrane systematic review of randomized trials in which nicotine replacement therapy was compared to placebo or no treatment or where different doses were compared	132 trials were identified for inclusion Nicotine replacement therapies increase the rate of quitting by 50–70%	No studies from Indigenous contexts identified
	Hughes et al. (41)	Cochrane systematic review of randomized trials comparing antidepressant medications to placebo or an alternative pharmacotherapy for smoking cessation	52 trials were identified for inclusion Bupropion and nortriptyline aid long-term smoking cessation, but selective serotonin reuptake inhibitors do not	No studies from Indigenous contexts identified
	Holt et al. (42)	Randomized, placebo-controlled, double-blind parallel group study of 134 Maori smokers who consumed more than 10 cigarettes per day	At 3-month follow-up, the rates of continued abstinence in the bupropion and placebo groups were 44.3 and 17.4%, respectively At 12-months, corresponding figures were 21.6 and 10.9%	Did not recruit the projected 141 participants required to detect difference in proportions between groups at an α value of 0.05 with 80% power
	Robles et al. (43)	Systematic review of studies evaluating the use of smoking-cessation pharmacotherapies in non-white populations in USA	Nine studies were identified for inclusion (six related to black smokers, one related to Hispanic smokers, one related to Native American smokers, and one related to a group of white and non-white smokers) For smokers in the Native American study (*n* = 252), self-reported abstinence rates were 31% (49/156), 30% (21/71), 24% (13/55), and 21% (4/19) at 3, 6, 9, and 12 months, respectively	Potential for response bias in Native American study
	Campbell et al. (44)	Community-based tobacco intervention trial conducted over 1 year in five intervention sites and three control sites in predominately Indigenous communities in North Queensland Intervention comprised six components (school education, brief intervention training, assistance to develop smoke-free workplace policies, event support program, support group, and enforcement of tobacco sales restrictions) Household surveys of self-reported tobacco use conducted at baseline and 1-year post-intervention	Modest effect on self-reported daily tobacco use and mean number of cigarettes smoked weekly at follow-up in intervention communities; non-significant declines in control communities	Potential for response bias Impact of pharmacotherapy relative to other components of the intervention not clear
	Richmond et al. (45)	Prospective study in a maximum security prison A total of 31 participants (Indigenous and non-Indigenous) received two brief cognitive behavioral therapy sessions, nicotine replacement therapy, bupropion, and self-help resources Biomedically validated point prevalence and continuous abstinence measured at 6-month follow-up	At follow-up point, prevalence was 26% and continuous abstinence rates were 22% Those who relapsed or continued smoking smoked less than at baseline	Small sample size
	Ivers et al. (46)	Pre- and post-test interviews with 34 Indigenous smokers who self-selected to receive free nicotine patches and a brief intervention, and a further 59 who chose to receive brief intervention only (receiving advice, viewing a flip chart, and being offered a pamphlet)	At 6-month follow-up, 15% of the nicotine patch and brief intervention group reported that they had quit smoking (10% with biochemical validation) compared to 1% (biochemically validated) of the group that elected brief intervention only	Small sample size Participants assigned to groups through self-selection
	Karen et al. (47)	Prospective study in which 32 Indigenous individuals were exposed to a multicomponent intervention providing the opportunity to sign up for Quitline and receive weekly phone calls from the service, access to nicotine replacement therapy and Zyban, general practitioner consultations, and access to a Quit facilitator and educator	19% quit rate	Impact of pharmacotherapy relative to other components of the intervention not clear Small sample size
Indigenous-specific social marketing campaigns	Murphy and Mee (28)	15 focus group discussions with 42 Indigenous adults, 20 Indigenous teenagers, and 23 community and health workers recruited across four locations	Found that “the groups gave no indication that awareness of, or exposure to, the campaign was any different among indigenous and non-indigenous populations. Similarly, the results … gave no reason to believe that indigenous people received the campaign messages any differently to the non-indigenous population”	Evaluation contained no description of methods by which participants were recruited and data were analyzed
	Ivers et al. (48)	351 (mostly Indigenous) people from a source population of 1,228 residents participated in a community survey at both baseline and 12-month follow-up Populations were exposed to multicomponent tobacco control interventions, including advertising	86% of smokers recalled seeing non-Indigenous anti-tobacco advertisements 10 people who claimed to be smokers at baseline visit had quit at follow-up Those who recalled seeing the advertisements were not significantly more likely to quit than those who did not Logistic regression showed that exposure to individual tobacco interventions was not associated with an increased chance of cessation during intervention year	N/A
	Wilson et al. (49)	Monthly Quitline call data and calls within 1 h of a television commercial being shown were analyzed for 2002–2003 Data on target audience rating points (TARPS) also used	Mainstream commercial generated 115 calls per 100 TARPS from Maori callers within 1 h of airing Maori-oriented commercial generated 91 calls per 100 TARPS from Maori callers within 1 h of airing	Did not measure actual impact on tobacco cessation Two campaigns not directly comparable as mainstream campaign was focused on tobacco control only, while Maori-oriented campaign included broader messages about health, well-being, and cultural identity
Enhanced Quitline services	Karen et al. (47)	Prospective study in which 32 Indigenous individuals were offered a multicomponent intervention (see above)	19% quit rate Noted “a difficulty has been people expressing apprehension about receiving support through enrollment with Quitline. With encouragement to receive one call and give it a try all participants have used Quitline with to date no negative complaints voiced about the service”	See above
	Maher et al. (50)	Telephone survey of 1,312 callers (including *n* = 101 Native American/Alaskan, *n* = 762 white) to a Quitline 3 months after their initial call Analysis compared 7-day quit rates and satisfaction measures by race/ethnicity and other factors	Seven-day quit rate was higher among Native American/Alaskan callers (35%) 3 months after the initial call, compared to white callers (30%) (*p* = 0.42) Native American/Alaskan callers reported high rates of service satisfaction	Not clear whether any difference in response rates between Native American/Alaskan and white callers
	Hayward et al. (51)	Comparative study of utilization and effectiveness of Canadian quitlines among first-time callers who completed an evaluation and provided ethnic status (*n* = 7,082)	A 6-month prolonged abstinence rate for Aboriginal men was 16.7% compared with 7.2% for Aboriginal women and 9.4 and 8.3% for non-Aboriginal men and women, respectively	Findings cannot be assumed to be representative given that they are based on responses from only those who provided ethnic status and agreed to participate in the evaluation

### Rationale for Adopting Policy Proposals in the Absence of Strong Evidence

It is clear from Tables 3 and 4 that not all of the policy initiatives were grounded in strong evidence demonstrating their likely effectiveness. A critical analysis of the studies revealed that many had limitations, such as inadequate reporting of methods, selection bias, response bias, and small sample sizes. Most Indigenous Australian studies measured subjective preferences for interventions, perceptions of *likely* impact of interventions, or thoughts and attitudes about smoking. In the absence of rigorous evidence from the Australian context, some reliance was placed on studies of indigenous populations in other countries. Interviews were used to understand why the policy makers adopted the policy proposals in the absence of clear supporting evidence.

#### Impractical to Wait for Rigorous Indigenous-Specific Evidence

Faced with both a dearth of evidence and an understanding that Indigenous smoking rates were inexorably high despite the presence of mainstream tobacco control strategies, some policy makers saw the need to take action based on inference. An Indigenous tobacco control academic with close links to key policy makers noted that *“the problem is we didn’t have any evidence from this [Indigenous] setting so* … *people had to make guesses. And they probably weren’t bad guesses.”* A Tobacco Working Group member used similar language, describing the research conducted to develop policy priorities as *“predominately guess-based.”*

An innovate and evaluate approach was accepted as being an appropriate solution in the circumstances. Internal documents obtained under FoI demonstrate the extent to which policy makers considered that it was both necessary and justifiable to act without a strong evidence base, so long as there was a commitment to accumulating knowledge in the process:

Currently there is little available evidence for what works in Indigenous tobacco control. In partnership with research bodies … the Government can make an immediate start on building that evidence-base …

In tandem with the research program, it is proposed to run five or six pilot projects in selected locations to trial innovative approaches to smoking prevention and cessation … Evaluation of these pilots could inform future phases of the initiative …

The approach recommended here combines action (the pilot programs) coupled with a strong research focus to ensure that resources are not wasted on misdirected initiatives.^2^

The alternative approach – that is, waiting for evidence to emerge before taking policy action – was not considered practical. Two policy makers noted that the Indigenous context presented distinct challenges to the conventional understanding of evidence-based policy making, arguing that evidence needed to play more of a back-end role. According to a Tobacco Working Group member:

*[W]e need to look at ways of finding types of intervention that would resonate better with Aboriginal communities and I think a part of what came with that [was] also an acceptance that certainly when it comes to mainstream programs, it is driven by research* … *but* … *when it comes to Aboriginal smoking there’s a need to be willing to take a few more risks, and trial different ways of addressing the problem*.

Researchers with links to policy makers expressed the view that the Indigenous research context presented distinct challenges. One respondent offered the following example of the limitations:

*[T]he logistics of doing high level randomised controlled trials in [remote] populations* … *it’s ridiculous* … *There were a whole lot of issues with cross-contamination between communities* … *We did the numbers for a randomised control trial in giving quit advice* … *the numbers to do a proper randomised control trial in the Northern Territory were going to be 30,000 participants. You couldn’t get it* … *(Indigenous tobacco control academic)*.

An Indigenous advocate with research experience added:

*[T]here’s always challenges from the remoteness of going out to do the pre- and post-questionnaires* … *a lot of these people move around in their communities* … *They will travel around and visit family and the neighbouring community. Could be anything* … *even the weather* … *From all those kind of logistic things, to even language being a barrier*.

#### Policy Proposals Were Championed by Indigenous Representatives

Not all members of the policy community subscribed to the view that mainstream tobacco interventions were ineffective for Indigenous people, or that the Indigenous-specific interventions recommended in the CEITC paper were needed. One Tobacco Working Group member warned against *“overdoing the differences”* and noted that *“Aboriginal people are people. They smoke like other people. They quit like other people. And I think we need to be almost wary of assuming that you need to have something incredibly and dramatically different.”* Another Working Group member concurred, arguing that *“there is absolutely zero evidence that those [mainstream] approaches do not impact Indigenous people in the same sort of was as they affect the rest of us.”* Longitudinal studies lend some support for this view showing that, in the absence of major Indigenous-specific policies, daily smoking prevalence for Indigenous males over the age of 18 years decreased from 58.5% in 1994 to 52.6% in 2008 with a statistically significant annual decrease of 0.4% per year (52).

The apparent mismatch between the advice provided to Government and the view of some respondents that some Indigenous-specific interventions were not necessary was explained in the following way:

*I know there would have been at least two or three people around the table who would have shared what I just said to you about ‘oh, god, do we really have to go down this* … *culturally distinctive route?’ But I think in the scheme of things we didn’t feel that strongly about it to say, ‘listen, let’s just can that.’ Because I think that critique needs to come out of the Indigenous community rather than be given like Moses from above”* (Tobacco Working Group member).

An expert government advisor agreed that, in light of the history of exclusion, Indigenous perspectives need to be incorporated into the policy process:

… *[P]eoples outside of mainstream Australia have a right and should be involved in leading the progression of policy development around those marginalised populations* … *[I]t’s time for the policy developers in Australia to now say to Aboriginal Australians, ‘you must develop the policies and you must assist us on the pathways out’* (National Indigenous Drug and Alcohol Committee member).

Other members of the Tobacco Working Group confirmed the importance of ensuring that the policies were developed with and supported by Indigenous people:

*[B]ecause of the awful history for Aboriginal people in this country there’s a real strong desire amongst representatives of the Aboriginal community that you’ll be working with* … *wanting it to be Aboriginal-determined, controlled etc*.

*[A] lot of Aboriginal people were really keen on the idea of local workers. Would it be in my core program? Not necessarily. But if it’s the price we pay for getting Aboriginal people [to] support the rest of the program – willingly pay it*.

## Discussion

This case study has demonstrated empirically what the theoretical models of policy making suggest – namely – that evidence does not exist and cannot be used in a vacuum. Rather than viewing evidence as being in *competition* with other policy factors, this case study presents evidence as being *dependent* on them.

The intuitive appeal of evidence-based policy making lies in the perception of objectivity – evidence “speaks for itself” (53). Certainly, the data suggested that evidence played an important role in getting Indigenous smoking onto the political agenda. The salience of the Vos burden of disease study was repeatedly described in interview responses and relevant documents (17). However, the findings show that while “[s]cience may be able to deliver empirical knowledge … this knowledge has to be interpreted and warranted in order to be understood” (54).

In this instance, there were two factors that enabled the evidence to “speak” in the policy process. First, the evidence emerged against the backdrop of a popular sociopolitical campaign to reduce health disparities between Indigenous and non-Indigenous Australians; this aligns with both Walt and Gilson’s notion of “context” and Lin’s definition of “cultural rationality” as “what (perceived) societal opinions feel is right” (3, 5). Second, the evidence was understood and valued by key figures in Government (including the Minister for Health) who had the power to take action in response; this reflects the importance of “actors” and “process” in Walt and Gilson’s model, and “political rationality” in Lin’s schema (3, 5).

The symbiotic relationship between evidence and other policy factors was further demonstrated in the policy design stage. The recommendations presented in the government authored/commissioned literature reviews (obtained under FoI) seem to have heavily influenced the content of the policies. In relation to the *Indigenous Tobacco Control Initiative*, “political rationality” and “context” seem to be major explanations for the fact that most of the recommendations emerging from the literature review were adopted in the policy. The political pressure to make a major policy announcement at the Indigenous Health Equity Summit meant that policy proposals needed to be developed by DoHA within a short period of time. In this situation, the importance of the evidence seems, at least in part, to have been associated with its accessibility (the literature review was drafted internally by the Department in the previous year). The accessibility of research has been identified as a facilitator to evidence-based policy making in other studies (55–58).

Similarly, the *Tackling Indigenous Smoking Measure* closely reflected the recommendations in another literature review commissioned by DoHA and authored by the CEITC. Viewed through the prism of Walt and Gilson’s model of health policy making, the value that was placed on the CEITC’s research can be partly ascribed to the organization being regarded as a key “actor” in the policy community. If the policy community is conceptualized as a network of interconnected individuals and organizations, CEITC’s influence derived from its centrality in that network (59). DoHA provided CEITC with a majority of its funding with a view to building the evidence base in relation to Indigenous tobacco control and, as demonstrated in the interview data, relied on the organization for policy advice. Furthermore, the Director of CEITC was a member of the Tobacco Working Group, which was also consulted in the development of the policy. From the perspective of Lin’s model, “political rationality” overlapped with “technical rationality” in the sense that a research organization was invited into the decision-making process.

While it is clear that the tobacco control policies were developed based on evidence in the form of literature reviews, a critical analysis of the studies contained in those literature reviews revealed that many possessed methodological limitations. The evidence relied on was equivocal as to whether the recommendations would lead to positive tobacco control outcomes for Indigenous Australians. The case study therefore highlights the importance of conceptual clarity when using the term “evidence-based policy.”

However, it does not follow that policies based on weak or equivocal evidence are “bad policies.” Far from being irrational or reckless, the interview data suggest that the policy makers’ acceptance of the policy proposals was based on logical reasoning, notwithstanding the absence of a rigorous evidence base.

Respondents noted the importance of other influences on the policy process, including the need to ensure that the policies had the support of members of the Indigenous population. Historically, policy making for Indigenous Australians has often been a top-down exercise associated with marginalization and disempowerment of the population. For instance, an inquiry into a controversial 2007 Indigenous policy initiative (the Northern Territory National Emergency Response) found that the policy “diminished its own effectiveness through its failure to engage constructively with the Aboriginal people it was intended to help” (60). For some of the policy makers in the present case study, there was a desire to avoid repeating mistakes of the past by ensuring that the needs and preferences of Indigenous people were captured in the design of the *Indigenous Tobacco Control Initiative* and *Tackling Indigenous Smoking Measure*.

Evidence cannot trump all other factors; rather it must coexist with other inputs in the policy process, including the “thoughtful identification and compassionate use of individual patients’ [or populations’] predicaments, rights and preferences” (61). This can be illustrated by another example from the Australian Indigenous health context. While there is a strong evidence base to support the use of dialysis for patients with renal failure, historically, many Indigenous patients living in remote areas refused treatment on the basis that it would involve spending extended periods of time in urban and regional centers, away from their family, culture, and land (62). Put simply, “an efficacious treatment cannot produce public health benefits if it is not adopted widely by its intended recipients” (63).

Ideally, solutions that are “acceptable” to the target population will also be supported by evidence of their likely effectiveness. However, the Indigenous tobacco control case study demonstrates that there are situations in which it may be justifiable to introduce policies in the absence of high-quality evidence.

It has been noted that barriers to research in Indigenous contexts include

- the fact that the Indigenous Australian source population is small (670,000), meaning that sample populations in Indigenous-specific studies are also likely to be small, and only very large program impacts are likely to be statistically significant (64);
- Indigenous distrust of research on the basis that it was historically associated with “the politics of colonial control” and, in the present day, can be perceived to be exploitative, stigmatizing, or unresponsive to Indigenous needs and concerns (65–67); and
- the fact that, in order to be culturally sensitive, there is often a call for research in Indigenous communities to be participatory, which can result in a conflict between “the values of the academic setting and those of the community” (65, 68).

The consequence is that “‘best’ evidence is often gathered on simple interventions and from groups that are easy to reach in a population,” and evidence from those that are harder to reach tends to be regarded as inferior (69).

This research bias is not unique to the Indigenous Australian context. It has been noted that low-income countries often have limited resources to invest in research, and cultural minorities within high-income countries are often excluded from research protocols because of the logistical and financial costs, such as those related to translation (70). With respect to tobacco control research, a systematic review of studies examining the effectiveness of NHS stop smoking services in the United Kingdom found there is currently a lack of high-quality research into the potential differential impact of interventions in other subpopulations, such as black and minority ethnic groups, despite qualitative and anecdotal evidence suggesting different beliefs and attitudes to smoking and smoking cessation (71).

Birch presents the potential resulting paradox of a “no action without supporting evidence” mentality thus:

If there is no evidence of effectiveness among the most deprived group… [e]fficient use of scarce resources… would imply the intervention not be given to these individuals with the anticipated increase in social inequalities in health. The resources of a health care system would become increasingly concentrated on less deprived populations. This results not necessarily because the problems of the more deprived groups are insoluble, but because the mechanisms we choose to evaluate for dealing with the observed problems are particularly suited to less deprived groups, i.e. it is a feature of the ‘service focus’ (72).

Applied to the Indigenous tobacco control context, a rigid adherence to the belief that there should be “no policy without evidence” could feasibly have resulted in a widening of the health gap between Indigenous and non-Indigenous Australians simply because there was more research to demonstrate the effectiveness of interventions in the latter population.

When the potential corollaries of the “no policy without evidence” approach are laid bare, the policy makers’ decision to adopt the *Indigenous Tobacco Control Initiative* and *Tackling Indigenous Smoking Measure* in the absence of strong evidence is justifiable. The policies were not made with reckless disregard for what would work; instead, evidence from other settings and expert opinions were used to generate conceptually plausible responses to the policy problem in a manner that accommodated the apparent preferences of members of the Indigenous community. Moreover, the policy proposals were accompanied by a commitment to evaluate their effectiveness. This approach is consistent with that advocated by Sanderson, who suggests that policies should be viewed as hypotheses to be tested through pilots or trials and/or rigorous monitoring and evaluation (73).

In May 2015, the Government committed a further AUD116.8 million to Indigenous tobacco control initiatives over 3 years (74). Future research needs to examine whether rigorous evaluations of the *Indigenous Tobacco Control Initiative* and *Tackling Indigenous Smoking Measure* were conducted as planned and, if so, the extent to which lessons learnt have informed more recent policy developments.

In terms of theories of the policy process, this case study has provided clear evidence of the role of “context/cultural rationality” and “process/political rationality” as having at least as much weight as “technical rationality” in understanding how this particular policy area evolved.

Furthermore, as this case study demonstrates, public health policy making does not always lend itself to the literal translation of rigorous research findings. More empirical case studies are needed to explore the role of other factors – such as the needs and preferences of the target population – in order to provide a clearer understanding of their relative importance in the policy process.

## Author Contributions

All authors made substantial contributions to the conception and design of the work. DV conducted data acquisition and initial analysis. DV drafted the initial manuscript, and MR, RF, and SA made critical revisions for important intellectual content. All authors approved the version to be published and agree to be accountable for all aspects of the work in ensuring that questions related to the accuracy or integrity of any part of the work are appropriately investigated and resolved.

## Conflict of Interest Statement

Nil. Please note that, pursuant to Australia’s federal system of government, the Western Australian Department of Health (institutional affiliation for the first named author) is a separate entity to the Commonwealth Department of Health and Ageing.

## Footnotes

^1^Department of Health and Ageing. *Tobacco Control and Aboriginal and Torres Strait Islander Australians. Internal Document Obtained under Freedom of Information Act 1982*. (2007).

^2^Anonymous. *Announcement of the Indigenous Tobacco Control Initiative at the Close the Gap Indigenous Health Equality Summit 20 March 2008. Internal Document Obtained under Freedom of Information Act 1982*. (2008).
